# Congruency of Ultrasound Measure Changes Against Patient-Reported Outcome Measures in Patellar Tendinopathy: A Systematic Review and Meta-Analysis

**DOI:** 10.7759/cureus.74200

**Published:** 2024-11-22

**Authors:** Cameron Parks

**Affiliations:** 1 Trauma and Orthopaedics, Manchester Royal Infirmary, Manchester, GBR; 2 Trauma and Orthopaedics, Queen Mary University of London, London, GBR

**Keywords:** injury, patellar, tendinopathy, ultrasound, visa-p

## Abstract

Patellar tendinopathy is a highly prevalent clinical diagnosis supported by ultrasound changes. Numerous interventions are targeted at improving both symptoms and structure of dysfunctional tendons, however little is known of the diagnostic value in a changing ultrasound profile whilst patient-reported outcome measures (PROMs) determine recovery. The aim is to assess if changes in ultrasound measures are congruent with changes in Victorian Institute of Sports Assessment - Patella (VISA-P) scores, thereby supporting the use of ultrasound to assess patellar tendinopathy during symptom improvement. Four databases (PubMed, Web of Science, Embase, CINAHL) were searched in January 2019. Studies selected contained ultrasound and VISA-P scores from ≥ 2 type points. All included studies were quality assessed depending on the type and available data and underwent meta-analysis. A total of 10 papers of varying study types, limited to high quality, were synthesized. The meta-analysis indicated that changes in ultrasound measures were not congruent with changes in the VISA-P score. The variation in study quality, along with significant heterogeneity of ultrasound measure outcomes and reporting may influence the congruency of the data, but the association between gradual structure change and varying vascularity with pain or function is questionable throughout tendinopathy literature. The small study yield coupled with omission of data, along with alterations to form coherent analysis may impact outcomes, with an absence of homogeneity throughout studies. Ultrasound assessment is not useful in the follow-up of patellar tendinopathy after treatment and is of limited use as an indicator of normal function.

## Introduction and background

Patellar tendinopathy, also known as jumper’s knee, is typically an overuse injury in athletes most commonly characterized by localized pain of the patella that increases during movements which increases anterior knee pressure and recruits the knee extensor mechanism [1]. Primarily a condition of young athletes between the ages of 15-30 years old, it is a highly prevalent condition, reported at 14.2% for elite and 8.5% for recreational athletes, which can cost one-third of athletes greater than six months out of their sport [2-4]. 

Imaging is a valuable skill in the clinical assessment of tendinopathy, with ultrasound and MRI being employed to assess tendon structure. With the increasing popularity of ultrasound as a quick, cheap, and minimally invasive technique for doing this, practitioners employ this frequently due to its high sensitivity and accuracy [2,5].

Patient-reported outcome measures (PROMs) are a reliable and valid method of assessing tendinopathy, and Victorian Institute of Sports Assessment - Patella (VISA-P) scoring is commonly used for patella tendinopathy specifically for the same reasons. The questionnaire evaluates symptomatic responses, sports participation ability, and clinical severity, regularly being used in multiple studies as an accurate indication of tendinopathy severity [6,7]. 

Ultrasound imaging has assessed a number of structural variations within tendons, including tendon thickness, amount of neovascularization, and hypoechoic regions. As tendinopathy is primarily based on clinical diagnosis, these further structural assessments have been shown to indicate a fourfold increase in the risk of future tendinopathy in asymptomatic individuals [8]. The predictive aid these measures offer has been invaluable; however, very little is known as to how these predictive features may change during the course of patellar tendinopathy treatment, and if a relationship can be found to show improvement of tendinopathy beyond reduced pain upon examination or report. 

Tendon abnormalities identified by measures such as increased tendon thickness, increased colour Doppler flow indicative of neovascularisation and hypoechoic shadowing can be present in as many as 59% of asymptomatic individuals [9,10]. The nature of these features being present in “asymptomatic” individuals has led to the belief that changes cannot be used to indicate regression of tendinopathy post-intervention and clinical presentation is the only reliable method of assessing athletes for a return to sport [11, 12].

The aim of this review is to assess whether a relationship can be found between ultrasound outcomes and VISA-P over time after the administration of an intervention to populations with patellar tendinopathy and whether any congruency of change could indicate a measure for future assessment and staging of tendinopathy receiving treatment. 

## Review

Search strategy and study selection

The PRISMA guidelines were used to guide the format and reporting of this review [13]. The following databases were searched in January 2019: PubMed, Web of Science, Embase and CINAHL. The search strategy is included in Appendix 1.

All searches were performed independently by three reviewers: CP, AR and KV. Once duplicates were removed, all three reviewers independently assessed the studies following PRISMA guidelines, firstly by screening all titles and abstracts and then refining further by assessing the full texts. Where there was disagreement, decisions were reached by discussion and consensus.

Inclusion and Exclusion Criteria

Upon completion of the original search and agreement upon eligible studies, the resulting studies were then refined further individually for eligibility in reviewers' own specific reviews dependent on outcome measures present; this included: Ultrasound assessment and VISA-P assessment which was assessed at two time-points or more

All papers had a full-text assessment for eligibility within the focused review, resulting in a final selection of studies specific to the required review from the original general number which satisfied all outcome possible measures. 

The inclusion and exclusion criteria are included in Table 1.

**Table 1:** Inclusion and exclusion criteria for the study

	Inclusion criteria	Exclusion criteria
Study design	All study types assessing outcome measures of patellar tendinopathy.	Unpublished material (PhD/MSc thesis), letters to the editor, reviews and conference abstracts.
Participants	Human subjects aged ≥18 years with patellar tendinopathy.	Animal, cadaver and in vitro studies. Studies assessing tendinopathy in other joints/regions, unless patellar tendinopathy patients were part of the study and results for this subgroup can be extracted separately.
Outcome	Papers assessing any of the nine core outcome domains: patient rating overall condition; participation; pain on activity; function; psychological factors; physical function capacity; disability; quality of life; pain over a specified time	Studies assessing outcomes other than listed.
Language	English	Non-English papers

Data extraction and meta-analysis

Data was extracted by two reviewers: CP and AR, and this included patient demographics, number of tendons assessed, intervention employed, ultrasound measures investigated, definition of how the ultrasound measure was scored, study type and results. 

Meta-analysis was performed on all papers with pre- and post-ultrasound data, along with VISA-P scores before and after the interventions. Means and standard deviation of the data were extracted where available to calculate Cohen’s d and standardised mean differences. RevMan (The Cochrane Collaboration, London, UK) was utilised and then Excel (Microsoft Corp., Redmond, WA) was employed to present the data as a scatter graph in order to assess the association of the data. The SMD point estimates were interpreted as minimal≤0.2; small=0.2-0.49; medium=0.50-0.79 and large≥0.8 [14]. As the change in each outcome measure - from pre-intervention to post-intervention - was compared, rather than between each of the outcome measures themselves, effect sizes were not calculated. If data was insufficient, authors were contacted, and in the event of no data provided, these were analysed descriptively. 

Quality Assessment

Due to the variety of study types included, different quality assessment tools were employed. For randomised control trials, the PEDro scale was used to assess their quality [15]. This 11-part tool provided score values for quality based on each criterion being clearly satisfied within the study. A score of 5 or over indicated that a study was of high quality, and below this indicated low quality, however, bias was assessed quantitatively using scores of 2, 3, and 5-7. This was because these criteria were thought to be integral in the regulation of study bias, whereas all others were employed solely for the comprehensiveness of reporting. Studies were ordered on their PEDro score but the level of bias was indicated separately. 

For the prospective case series, the STROBE criterion was employed [16]. This initiative checklist was developed to indicate the inclusion criteria for a high-quality observation study, with a checklist of 22 items to identify. A criterion was fulfilled if all aspects mentioned within were present, providing a score out of 22. The study quality was determined by the assessor. 

For all other non-randomized studies, the Downs and Black scale was employed to assess study quality [17]. This commonly used 26-feature checklist categorizes a score of 21 or more as high quality, 14 to 20 as moderate quality, and 7 to 13 as limited quality, with less than 7 being poor quality. Bias was assessed quantitatively using criteria 5, 11-15, 19, and 21-25 as these are integral features in the regulation of study bias. Criteria 27 is sometimes present; however, it was omitted due to ambiguity [18].

All ratings of methodological data were carried out independently by two researchers (CP and AR) with any resulting disagreement solved through discussion and consensus.

Results

Identification of Studies

A total of 11,179 results were yielded in total by the formal systematic database searches dated January 2019. The inclusion time period was between January 2009 - January 2019 due to the advancement in accuracy, validity and reliability of ultrasound imaging in the last decade, favouring high-quality data extraction from included studies. The PRISMA flow diagram is included for reference.

**Figure 1:**
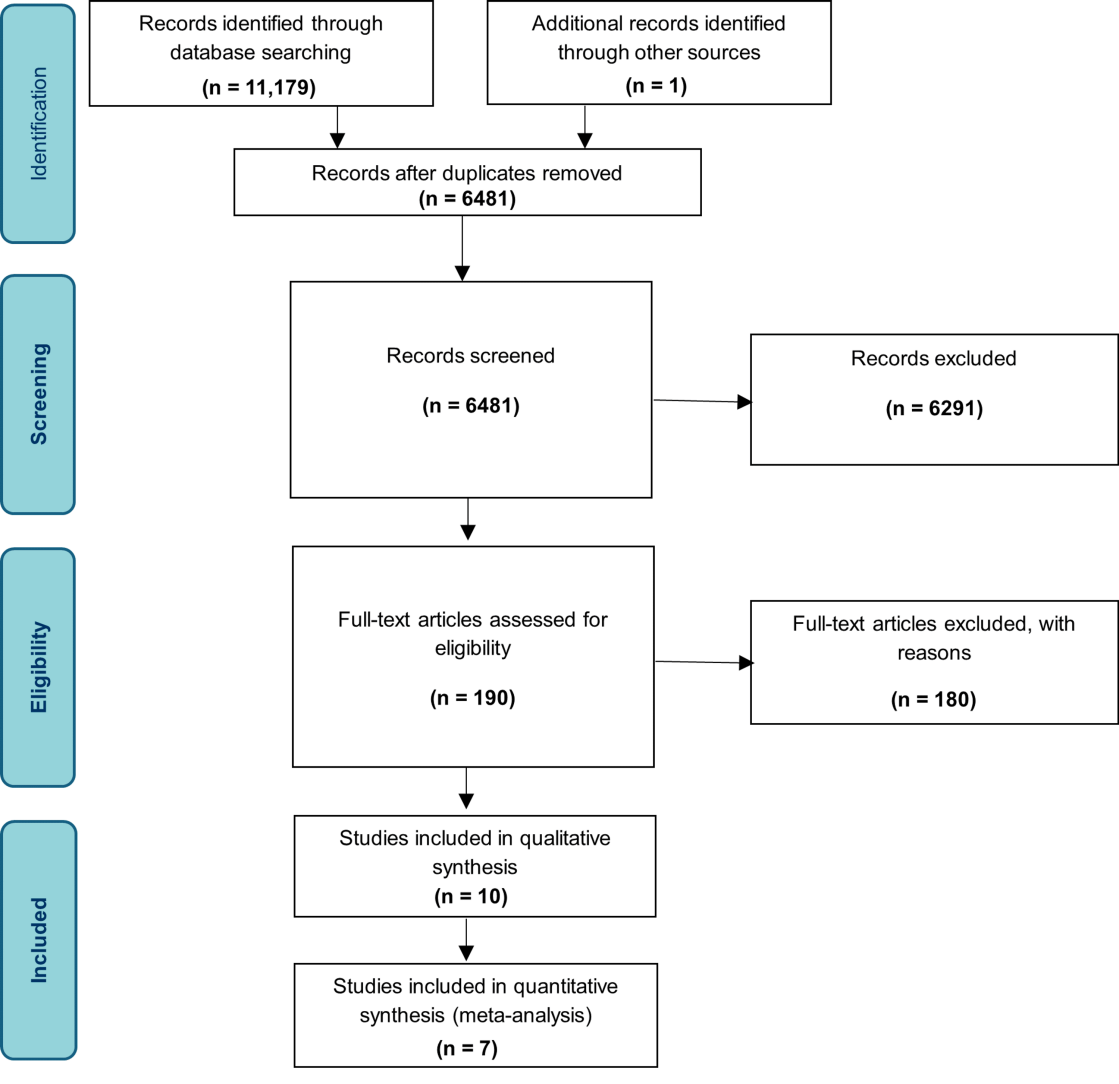
PRISMA flow diagram

Data Extraction

Illustrated in Table 2, participant sample sizes ranged from 11 [19] to 54 [20] and the total number of participants was 275, with tendon sample sizes varying from 14 [19] to 60 [21] when provided due to the presence of participants with bilateral tendinopathy. 

Study Characteristics

Seven of the trials assessed an intervention within only patellar tendinopathy populations [11,20-25], one assessed only Achilles and patellar tendinopathy [26], one Achilles, patellar and lateral epicondyle [27], and one Achilles, patellar and wrist extensor tendinopathy [19]. All specified inclusion and exclusion criteria, except two which omitted exclusion criteria [22,23]. All required a confirmed diagnosis of patellar tendinopathy via clinical and/or sonographic evaluation within inclusion criteria, with six requiring them to be unresponsive to previous treatment [19,21-24,26]. Only Van Ark et al. [11] stated a requirement of a certain level of exercise per week, as this is one of only two studies [26] which focused on an athletic population specifically - volleyball and basketball players. Exclusion criteria were much more variable, with the most common being systemic disease, such as rheumatic disease, featured in six studies [11,19,20,25-27]. Five trials did not exclude patients who had previous surgery, with five trials excluding patients with low haemoglobin or platelet count due to their use of patient-obtained PRP [20,21,23,24,26].

The summary mean age of the participants across the trails, calculated from the provided data, was approximately 33.5 years old. Of the 275 participants whose sex was defined, 79% were male and 21% were female.

**Table 2:** Characteristics of studies included SD: standard deviation; US, ultrasound; PRP, platelet-rich plasma; HVIGI, high-volume image-guided injection; SWE, shear wave elastography; B-Mode, brightness mode; mCSA, mean cross-sectional area; RCT, randomized controlled trial; CORT, corticosteroid; ECC: eccentric decline squat training; HSR: heavy slow resistance training

Authors	Intervention	Mean age (years old) ± SD	Mean BMI ± SD	Participants/Number of Tendons Assessed	Study Type	US Measures	Measure Definition
Gatz et al. 2017 [17]	Topical polidocanol + static stretching	26±5	Insufficient data	11(M) + 0(F) / 14; 7 left patella, 7 right patella	Prospective case series pilot	B-Mode + power Doppler + SWE	B-Mode assessed morphological changes, power Doppler graded via Öhberg scale, SWE evaluated quantitatively up to a max tissue rigidity 300kPa
Abate et al. 2018 [20]	PRP + HVIGI	HVIGI = 37±11.9; PRP= 38.6±12.4; HVIGI + PRP= 39.4±13.1	HVIGI = 24.5±2.9; PRP= 25±3.3; HVIGI + PRP= 25.5±3.3	HVIGI= 10(M) + 8(F); PRP= 7(M) + 11(F); HVIGI + PRP= 9(M) + 9(F)	Non-randomised intervention trial	US change + colour Doppler	US change defined by diffuse/focal tendon thickening, abnormal echotexture, ethesophytes and bone erosion. Colour Doppler graded according to semi-quantitative estimate of the number of vessels.
Van Ark et al. 2018 [11]	Isometric + isotonic exercise protocol	22.7±4.7	24.7±3.1	16(M) + 2(F)	RCT	Echo texture, thickness, mCSA of fibrillar structure	Echo texture graded by increasing intensity and distribution of grey areas into four types by algorithm – echo type 1 to 4
Arquer et al. 2014 [27]	Oral mucopolysaccharide + type I collagen + vitamin C	47.7±1.69	25.6±0.75	Insufficient data	Non-randomised intervention trial	Heteroechogenicity + hypoechogenicity + paratenon blurring + Neovascularisation	The percentage presence of varied or low echo response, effacement of the paratenon and percentage of vascular flow.
Clarke et al. 2011 [21]	Skin-derived tenocyte-like cells	36.0	Insufficient data	41(M) + 5(F) / 60; 27 left patella, 33 right patella	RCT	Overall thickness + hypoechogenicity + intrasubstance tears + colour Doppler neovascularity	Thickness of the tendon measured at the proximal tendon 5mm from the inferior patellar margin. Colour Doppler assessed via the number of colour pixels occupying the tendon origin graded 0 -10.
Ferrero et al. 2012 [26]	PRP	37.4	Insufficient data	14(M) + 10(F) / 28	Non-randomised intervention trial	Thickness + hypoechogenicity + power Doppler vascularity	Power Doppler assessed according to scale of 0=absent 1=few spots, 2=moderate, 3=intense.
Filardo et al. 2013 [22]	PRP	30.6±11.7	24.7±3.0	42(M) + 1(F) / 54	Non-randomised intervention Trial	Max thickness + colour Doppler vascularity	Max thickness in proximal third in sagittal plane view. All vessels >1mm totalled up for vascular length.
Kaux et al. 2016 [23]	PRP	PRP, 1= 31.1±10.4; PRO, 2= 29.5±5.87	Insufficient data	20(M) + 0(F)	RCT	Thickness + axial/sagittal hypoechogenicity + colour Doppler	Thickness defined as antero-posterior diameter. Colour Doppler defined as presence of a signal or not.
Kaux et al. 2015 [24]	PRP + submaximal eccentric protocol	28.8	Insufficient data	19(M) + 3(F)	Non-randomised intervention Trial	Echotexture + max tendon thickness + sagittal hypoechoic length + colour Doppler vascularity	Colour Doppler defined as presence of a signal or not.
Kongsgaard et al. 2009 [25]	CORT + ECC + HSR	CORT=34.3±10; ECC=31.3±8; HSR=31.7±8.5	CORT=24.8±2.2; ECC=24.4±2.1; HSR=24.8±3.2	COR=12; ECC=12; HSR=13	RCT	AP thickness + colour Doppler vascularity	Thickness measured 5mm distal from the apex of the patella. Colour Doppler defined as the total number of coloured pixels within the region of interest.

As seen in Table 3, follow-up varied from 20 days [26] up to approximately four years [22], but outcomes were most commonly collected at 12 and 26 weeks. Inexplicit reporting of study settings was common, but available information implied that the trials were conducted in hospital settings/outpatients.

Intervention Variation

Interventions and comparisons were varied across the studies. Six trials investigated the use of platelet-rich plasma (PRP), with one comparing HVIGI with a combination of PRP and HVIGI [20]. Another study by Clarke et al. [21] compared plasma alone against a combination of plasma and skin-derived tenocytes. One assessed its use with a submaximal eccentric protocol [24], and the three remaining assessed PRP injections over time with no comparison [22, 23, 26]. Other combination intervention studies included the use of topical polidocanol and static stretching by Gatz et al. [19], and the triple combination of oral mucopolysaccharide, type 1 collagen and vitamin C treatment by Arquer et al [27]. Van Ark et al. compared an isometric exercise protocol against an isotonic protocol to illustrate clinical improvements are not explained by changing tendon structure [11], whereas Kongsgaard et al. compared eccentric decline squat training and heavy slow resistance training programmes, along with corticosteroid injections [25] to assess their respective clinical effects.

Ultrasound Outcomes Assessed

The ultrasound outcome measures were increasingly diverse in comparison to the interventions. The most common was dimensional assessment of thickness, present in seven papers, in a variety of methods where specified. The most common was maximum tendon thickness [11, 22, 24], whereas Kongsgaard et al. and Kaux et al. opted to measure anteroposterior thickness instead [23, 25], and Clarke et al. chose a standardised 5mm distance from the proximal inferior patellar margin [21]. The next most common outcome measures were echotexture, again by varied definitions, and colour Doppler assessment for neovascularisation, both included in six papers each. Echotexture was defined by hypoechoic areas [21,23,24,26,27]. However, in the study by Van Ark et al., this was defined by the intensity and distribution of grey areas into four grades of increasing pathology - 1 to 4 [11]. The method with which colour Doppler was defined was much more variable, as Kaux et al. defined it as simply the presence of signal or not in both their papers [23, 24], Filardo et al. defined it as the total length of all vessels greater than 1mm [22], whereas Abate et al. assessed it is as a semi-quantitative estimate of the number of vessels present [20]. Comparatively, both Clarke et al. and Kongsgaard et al. defined it on the number of pixels present within the image, however, Clarke then translated this into a 0 - 10 scale [21,25]. Power Doppler was also used to assess vascularity [19,26], as well as numerous other methods used in single studies, which can be seen in Table 3. 

**Table 3:** Meta-analysis data HVIGI, high-volume image-guided injection; PROM, Patient Reported Outcome Measures; US, ultrasound; VISA-P, Victorian Institute of Sport Assessment-Patella; PRP, platelet-rich plasma; CI, confidence interval; SD, standard deviation

Study	Treatment	Ultrasound (US) measure	Group	PROM	Time (weeks)	n	Pre-Intervention US	Pre-Intervention US SD	Post-Intervention US	Post-Intervention Ultrasound SD	Pre-Intervention PROM	Pre-Intervention PROM SD	Post-Intervention PROM	Post-Intervention PROM SD	Cohen's D for US Measure	Cohen's D for PROM	Confidence Interval of Cohen's D for US Measure	Confidence Interval of Cohen's D for PROM	Corrected Scores
Abate et al. 2018 [20]	HVIGI	US change	Structure	VISA-P	12	18	2.1	0.8	1.9	0.8	52.8	9.1	65.7	11.5	-0.25	1.42	0.66	0.73	0.25
Abate et al. 2018 [20]	HVIGI	Colour Doppler	Vascularisation	VISA-P	12	18	3.2	0.8	2.4	1	52.8	9.1	65.7	11.5	-1	1.42	0.69	0.73	1
Abate et al. 2018 [20]	HVIGI	US change	Structure	VISA-P	26	18	2.1	0.8	1.8	0.8	52.8	9.1	63.4	9.8	-0.38	1.16	0.66	0.71	0.38
Abate et al. 2018 [20]	HVIGI	Colour Doppler	Vascularisation	VISA-P	26	18	3.2	0.8	2.3	1.2	52.8	9.1	63.4	9.8	-1.13	1.16	0.70	0.71	1.13
Abate et al. 2018 [20]	PRP	US Change	Structure	VISA-P	12	18	1.9	0.9	1.8	0.8	53.1	11.1	66.2	12.3	-0.11	1.18	0.65	0.71	0.11
Abate et al. 2018 [20]	PRP	Colour Doppler	Vascularisation	VISA-P	12	18	3.1	0.7	2.8	0.9	53.1	11.1	66.2	12.3	-0.43	1.18	0.66	0.71	0.43
Abate et al. 2018 [20]	PRP	US change	Structure	VISA-P	26	18	1.9	0.9	1.9	0.8	53.1	11.1	71.2	12.3	0	1.63	0.65	0.75	0
Abate et al. 2018 [20]	PRP	Colour Doppler	Vascularisation	VISA-P	26	18	3.1	0.7	2.3	1.2	53.1	11.1	71.2	12.3	-1.14	1.63	0.70	0.75	1.14
Abate et al. 2018 [20]	HVIGI + PRP	US change	Structure	VISA-P	12	18	2.2	0.8	2.2	0.9	51.9	8.5	72.2	14.3	0	2.39	0.65	0.86	0
Abate et al. 2018 [20]	HVIGI + PRP	Colour Doppler	Vascularisation	VISA-P	12	18	3.3	0.8	2.4	1	51.9	8.5	72.2	14.3	-1.13	2.39	0.70	0.86	1.13
Abate et al. 2018 [20]	HVIGI + PRP	US change	Structure	VISA-P	26	18	2.2	0.8	2.1	0.8	51.9	8.5	79.1	10.3	-0.13	3.2	0.65	0.99	0.13
Abate et al. 2018 [20]	HVIGI + PRP	Colour Doppler	Vascularisation	VISA-P	26	18	3.3	0.8	2.3	1	51.9	8.5	79.1	10.3	-1.25	3.2	0.71	0.99	1.25
Van Ark et al. 2018 [11]	Isometric + Isotonic (total pop.)	Antero-Posterior Diameter	Structure	VISA-P	4	18	7.8	1.3	7.5	1.9	67.5	14.1	81.5	10.9	-0.23	0.99	0.66	0.69	0.23
Clarke et al. 2011 [21]	Plasma alone	Overall thickness	Structure	VISA-P	26	27	8.513	2.282	7.517	1.822	50	18	70	14	-0.44	1.11	0.54	0.57	0.44
Clarke et al. 2011 [21]	Plasma alone	Hypoechogenicity	Structure	VISA-P	26	27	6.262	1.959	3.828	1.79	50	18	70	14	-1.24	1.11	0.58	0.57	1.24
Clarke et al. 2011 [21]	Plasma alone	Intrasubstance tears	Structure	VISA-P	26	27	8.613	4.977	1.822	1.686	50	18	70	14	-1.36	1.11	0.59	0.57	1.36
Clarke et al. 2011 [21]	Plasma alone	Colour Doppler	Vascularisation	VISA-P	26	27	2.304	2.754	2.353	2.149	50	18	70	14	0.02	1.11	0.53	0.57	0.02
Clarke et al. 2011 [21]	Skin-derived tenocytes + plasma	Overall thickness	Structure	VISA-P	26	33	9.146	1.666	7.913	1.368	44	15	75	17	-0.74	2.07	0.50	0.60	0.74
Clarke et al. 2011 [21]	Skin-derived tenocytes + plasma	Hypoechogenicity	Structure	VISA-P	26	33	6.52	1.584	4.13	2.302	44	15	75	17	-1.51	2.07	0.55	0.60	1.51
Clarke et al. 2011 [21]	Skin-derived tenocytes + plasma	Intrasubstance tears	Structure	VISA-P	26	33	10.47	7.146	2.414	2.194	44	15	75	17	-1.13	2.07	0.52	0.60	1.13
Clarke et al. 2011 [21]	Skin-derived tenocytes + plasma	Colour Doppler	Vascularisation	VISA-P	26	33	2.28	2.227	2.696	2.687	44	15	75	17	0.19	2.07	0.48	0.60	0.19
Ferrero et al. 2012 [26]	PRP	Thickness	Structure	VISA-P	2.86	28	17	8	16	9	56	18	60	19	-0.13	0.22	0.52	0.53	0.13
Ferrero et al. 2012 [26]	PRP	Thickness	Structure	VISA-P	26	18	17	8	11	5	56	18	74	14	-0.75	1	0.68	0.69	0.75
Filardo et al. 2013 [22]	PRP	Prox. third max. thickness	Structure	VISA-P	8	20	8.1	1.7	9.1	1.7	44.1	15.6	61.4	22.2	0.59	1.11	0.63	0.67	0.59
Filardo et al. 2013 [22]	PRP	Colour Doppler	Vascularisation	VISA-P	8	20	13.8	10.5	20	14.3	44.1	15.6	61.4	22.2	0.59	1.11	0.63	0.67	0.59
Filardo et al. 2013 [22]	PRP	Prox. third max. thickness	Structure	VISA-P	26	20	8.1	1.7	8.9	2	44.1	15.6	76.6	25.4	0.47	2.08	0.63	0.77	0.47
Filardo et al. 2013 [22]	PRP	Colour Doppler	Vascularisation	VISA-P	26	20	13.8	10.5	14.3	17.7	44.1	15.6	76.6	25.4	0.05	2.08	0.62	0.77	0.05
Filardo et al. 2013 [22]	PRP	Prox. third max. thickness	Structure	VISA-P	194	20	8.1	1.7	8.5	2	44.1	15.6	84.3	21.6	0.24	2.58	0.62	0.84	0.24
Filardo et al. 2013 [22]	PRP	Colour Doppler	Vascularisation	VISA-P	194	20	13.8	10.5	5.5	11.9	44.1	15.6	84.3	21.6	-0.79	2.58	0.64	0.84	0.79
Kaux et al. 2016 [23]	PRP	Antero-posterior diameter	Structure	VISA-P	6	10	9.1	1.9	8.4	1.5	46.3	13.35	54.4	19.62	-0.37	0.61	0.88	0.90	0.37
Kaux et al. 2016 [23]	PRP	Pathological: normal thickness	Structure	VISA-P	6	10	1.6	0.3	1.5	0.4	46.3	13.35	54.4	19.62	-0.33	0.61	0.88	0.90	0.33
Kaux et al. 2016 [23]	PRP	Axial hypo-echoic area	Structure	VISA-P	6	10	8.8	2.1	9.5	3.1	46.3	13.35	54.4	19.62	0.33	0.61	0.88	0.90	0.33
Kaux et al. 2016 [23]	PRP	Sagittal hypo-echoic area	Structure	VISA-P	6	10	17.4	4.8	25.6	11.2	46.3	13.35	54.4	19.62	1.71	0.61	1.02	0.90	1.71
Kaux et al. 2016 [23]	PRP	Antero-posterior diameter	Structure	VISA-P	12	10	9.1	1.9	8.5	1.8	46.3	13.35	63.4	22.71	-0.32	1.28	0.88	0.96	0.32
Kaux et al. 2016 [23]	PRP	Pathological: normal thickness	Structure	VISA-P	12	10	1.6	0.3	1.5	0.3	46.3	13.35	63.4	22.71	-0.33	1.28	0.88	0.96	0.33
Kaux et al. 2016 [23]	PRP	Axial hypo-echoic area	Structure	VISA-P	12	10	8.8	2.1	9.9	4.3	46.3	13.35	63.4	22.71	0.52	1.28	0.89	0.96	0.52
Kaux et al. 2016 [23]	PRP	Sagittal hypo-echoic area	Structure	VISA-P	12	10	17.4	4.8	28.7	11.7	46.3	13.35	63.4	22.71	2.35	1.28	1.14	0.96	2.35
Kaux et al. 2016 [23]	PRP	Antero-posterior diameter	Structure	VISA-P	6	10	8	1.7	9.6	1.3	46.1	11.55	54.8	21.36	0.94	0.75	0.92	0.91	0.94
Kaux et al. 2016 [23]	PRP	Pathological: normal thickness	Structure	VISA-P	6	10	1.8	0.2	1.6	0.2	46.1	11.55	54.8	21.36	-1	0.75	0.93	0.91	1
Kaux et al. 2016 [23]	PRP	Axial hypo-echoic area	Structure	VISA-P	6	10	12.4	6.3	13.1	6	46.1	11.55	54.8	21.36	0.11	0.75	0.88	0.91	0.11
Kaux et al. 2016 [23]	PRP	Sagittal hypo-echoic area	Structure	VISA-P	6	10	12.9	3.1	19.9	10.7	46.1	11.55	54.8	21.36	2.26	0.75	1.12	0.91	2.26
Kaux et al. 2016 [23]	PRP	Antero-posterior diameter	Structure	VISA-P	12	10	8	1.7	9.7	2.4	46.1	11.55	60.8	17.81	1	1.27	0.93	0.96	1
Kaux et al. 2016 [23]	PRP	Pathological: normal thickness	Structure	VISA-P	12	10	1.8	0.2	1.7	0.2	46.1	11.55	60.8	17.81	-0.5	1.27	0.89	0.96	0.5
Kaux et al. 2016 [23]	PRP	Axial hypo-echoic area	Structure	VISA-P	12	10	12.4	6.3	11.9	6	46.1	11.55	60.8	17.81	-0.08	1.27	0.88	0.96	0.08
Kaux et al. 2016 [23]	PRP	Sagittal hypo-echoic area	Structure	VISA-P	12	10	12.9	3.1	14.4	5.4	46.1	11.55	60.8	17.81	0.48	1.27	0.89	0.96	0.48
Kaux et al. 2015 [24]	PRP + submaximal	Pathological: normal thickness	Structure	VISA-P	6	20	1.6	0.2	1.5	0.3	47.9	17.9	61.1	20.4	-0.5	0.74	0.63	0.64	0.5
Kaux et al. 2015 [24]	Eccentric protocol	Max. tendon thickness	Structure	VISA-P	6	20	8	2.4	8	1.8	47.9	17.9	61.1	20.4	0	0.74	0.62	0.64	0
Kaux et al. 2015 [24]	Eccentric protocol	Sagittal hypoechoic length	Structure	VISA-P	6	20	15.3	7.14	19.5	10.9	47.9	17.9	61.1	20.4	0.59	0.74	0.63	0.64	0.59
Kaux et al. 2015 [24]	Eccentric protocol	Pathological: normal thickness	Structure	VISA-P	12	20	1.6	0.2	1.5	0.3	47.9	17.9	65.6	22.8	-0.5	0.99	0.63	0.66	0.5
Kaux et al. 2015 [24]	Eccentric protocol	Max. tendon thickness	Structure	VISA-P	12	20	8	2.4	8.3	1.9	47.9	17.9	65.6	22.8	0.13	0.99	0.62	0.66	0.13
Kaux et al. 2015 [24]	Eccentric protocol	Sagittal hypoechoic length	Structure	VISA-P	12	20	15.3	7.14	21.9	12.2	47.9	17.9	65.6	22.8	0.92	0.99	0.65	0.66	0.92
Kongsgaard et al. 2009 [25]	Corticosteroid	Antero-posterior thickness	Structure	VISA-P	12	12	7.3	2	6.3	1.8	64	14	82	19	-0.5	1.29	0.81	0.88	0.5
Kongsgaard et al. 2009 [25]	Corticosteroid	Colour Doppler	Vascularisation	VISA-P	12	12	11089	10433	6534	8497	64	14	82	19	-0.44	1.29	0.81	0.88	0.44
Kongsgaard et al. 2009 [25]	Eccentric	Antero-posterior thickness	Structure	VISA-P	12	12	7.3	1.3	6.6	1.3	53	13	75	3	-0.54	1.69	0.81	0.93	0.54
Kongsgaard et al. 2009 [25]	Eccentric	Colour Doppler	Vascularisation	VISA-P	12	12	11186	6607	8939	6276	53	13	75	3	-0.34	1.69	0.81	0.93	0.34
Kongsgaard et al. 2009 [25]	Heavy slow resistance	Antero-posterior thickness	Structure	VISA-P	12	13	8.3	2.2	7.1	1.7	56	13	78	18	-0.55	1.69	0.78	0.90	0.55
Kongsgaard et al. 2009 [25]	Heavy slow resistance	Colour Doppler	Vascularisation	VISA-P	12	13	15116	8749	9069	6447	56	13	78	18	-0.69	1.69	0.79	0.90	0.69

VISA-P was employed in all papers to assess the functional severity of patellar tendinopathy for comparison against the variable ultrasound methods as determined by the aim of this review, however, other PROMs were included in a number of the studies, such as VAS [20,23-25], IKDC [23,24], and Blazina and Tegner [22].

Meta-Analysis

As seen in Table 3, all mean values for the ultrasound measures and VISAP were collected as pre and post data, to formulate the Cohen’s d for each ultrasound measure and their respective VISA-P measurements from that study. Corrected scores for the difference in ultrasound were created in the final column in order to make the difference values comparable with the VISA-P change, as the trend is aiming to be identified not the data values within. 

**Figure 2:**
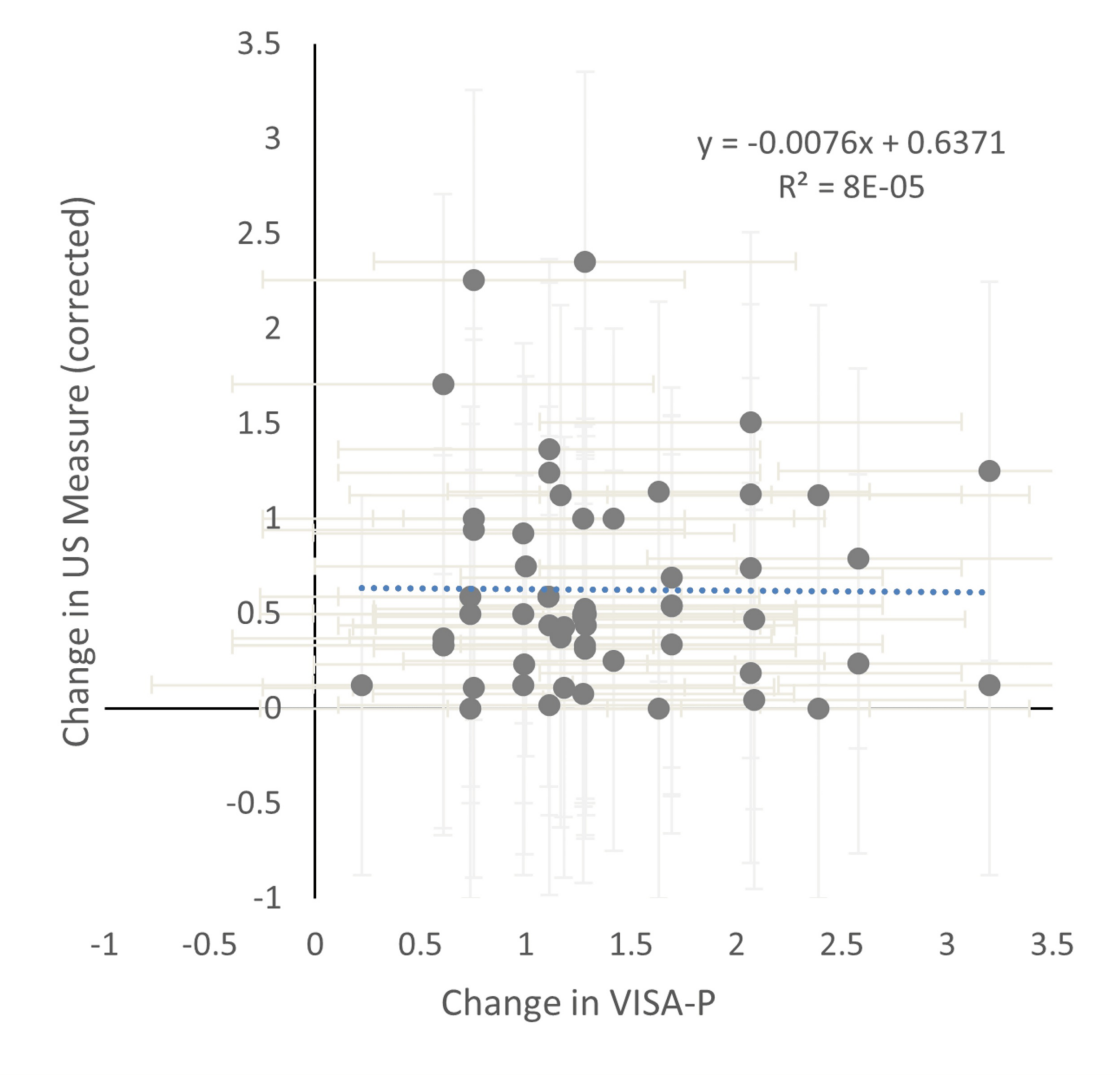
Change in ultrasound measure against change in VISA-P US, ultrasound; VISA-P, Victorian Institute of Sport Assessment-Patella

In doing so, it has shown the heterogeneity of the data produced when all ultrasound measures are compared against each other. Figure 2 exhibits all data points regardless of measure to show that there is no congruency between VISA-P change and ultrasound measure change.

**Figure 3:**
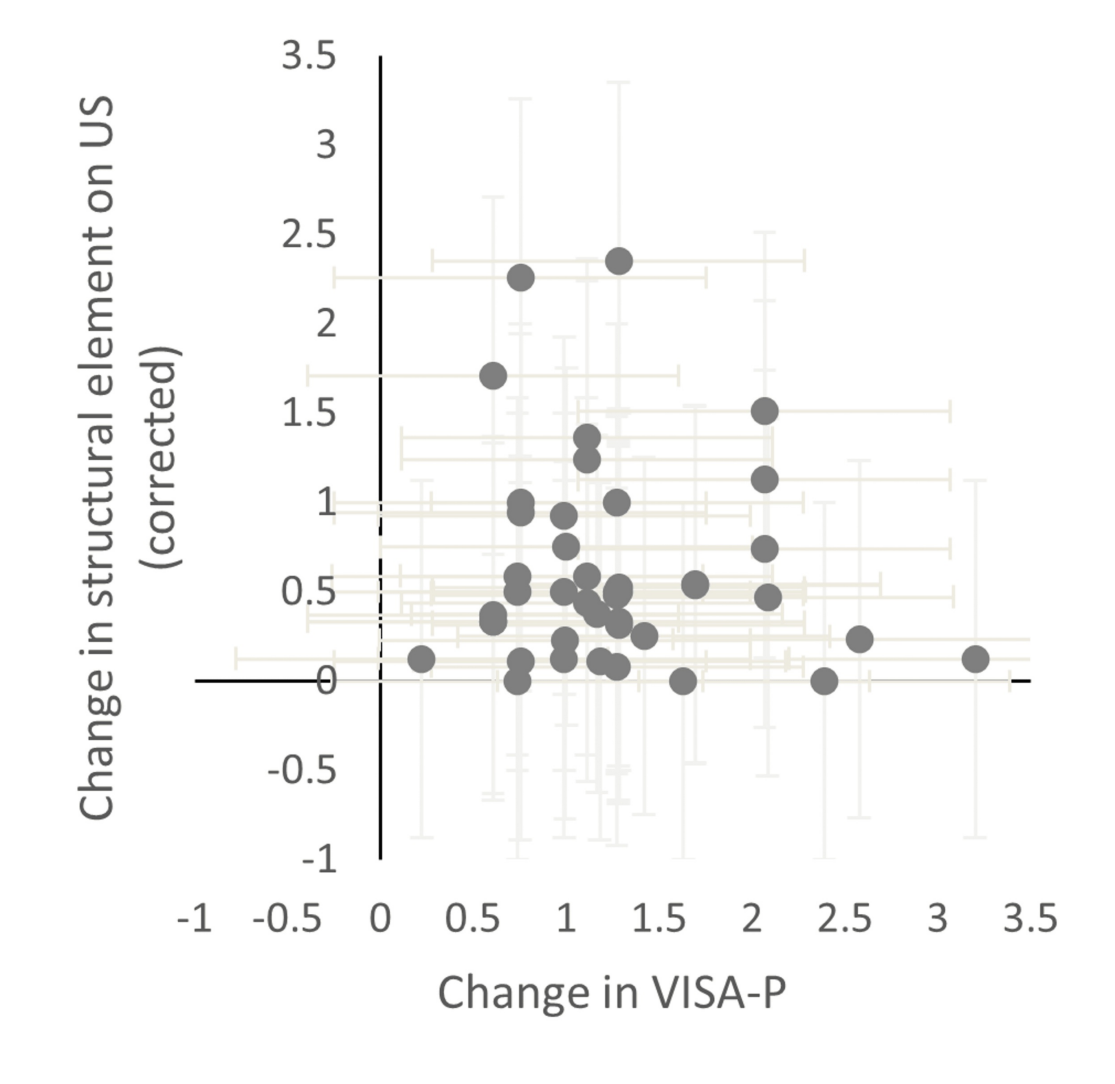
Change in a 'structural' element on ultrasound vs change in VISA-P US, ultrasound; VISA-P, Victorian Institute of Sport Assessment-Patella

**Figure 4:**
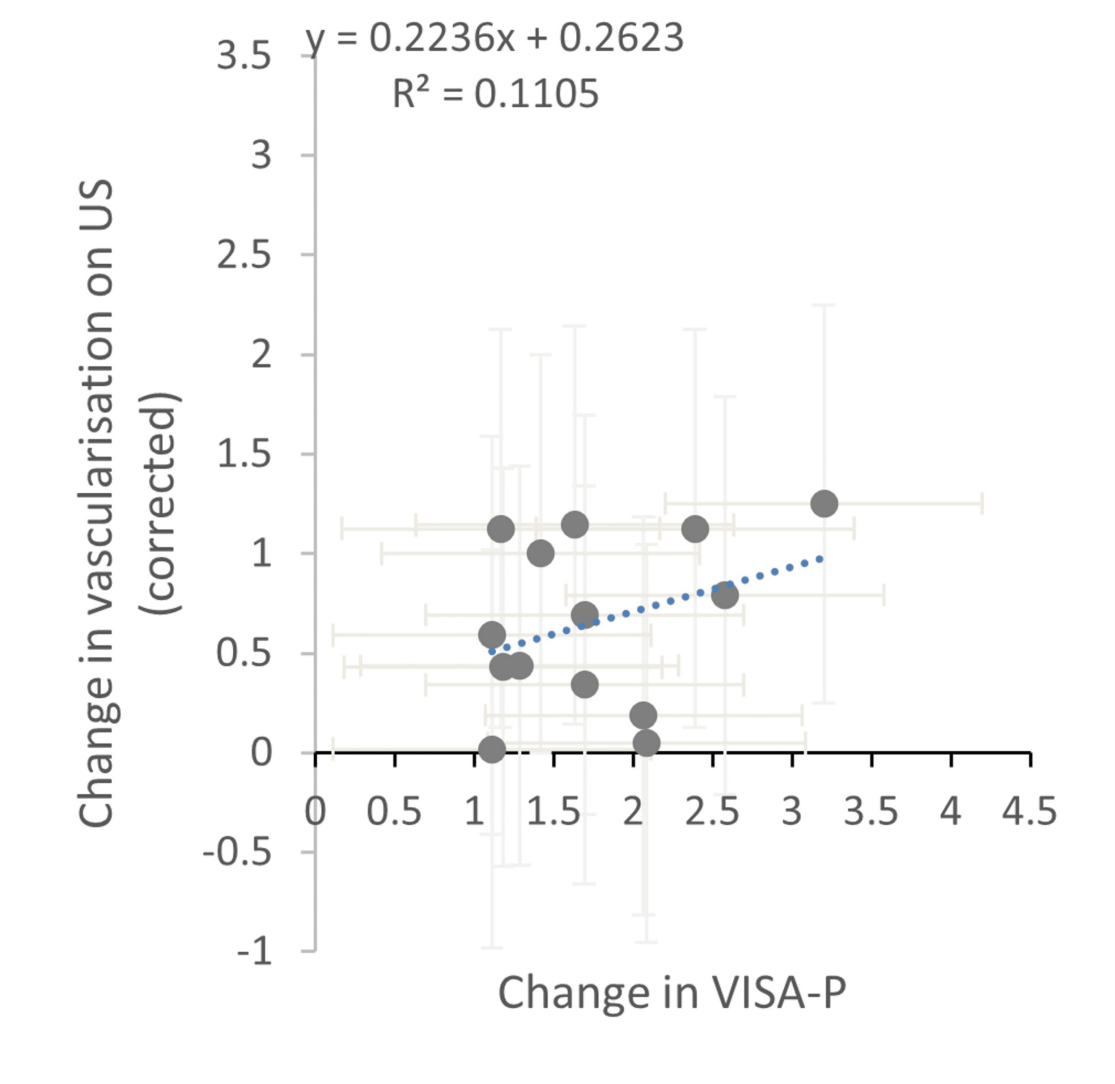
Change in vascularisation on ultrasound vs change in VISA-P US, ultrasound; VISA-P, Victorian Institute of Sport Assessment-Patella

Figures 3-4 show ultrasound measures split into structural elements and vascularisation. This further shows that structural change has no impact on VISA-P responses, and vascularisation data shows a limited but coherent trend. 

Due to insufficient data, a number of papers were omitted from the meta-analysis. Gatz et al. [19] described a moderate correlation between shear wave elastography mean (r=0.6; p=0.05) and max (r=0.42; p=0.2) with a VISA-P improvement of 13 points over six months. However, B-mode (r=0) and power Doppler (r=-0.5) showed no correlation. 

Arquer et al. [27] also showed an improvement in VISA-P of 46% over 90-day treatment but was only associated with tendon thickness change out of the ultrasound measures investigated. Within the paper by Van Ark et al. [11], US measures were broken down by intervention and also showed no significant change in improving VISA-P. Conversely, Ferrero et al. [26] also assessed hypoechoic areas and power Doppler, which exhibited significant reduction and increase respectively at six months. Though significant, an increase in vascularity with an increase in the VISA-P score is not expected as vascularity is hypothesised to be the factor in poor functional and pain results. 

Methodological Study Quality

Methodological quality was assessed using three different tools - four using the PEDro scale, five using the Downs and Black scale and one prospective case series using the STROBE statement. 

Using the PEDro tool for the four RCTs shown in Table 4, Item 2 (‘random allocation’), Item 9 (‘outcome analysis’), Item 10 (‘between group comparisons’) and Item 11 (‘point estimates and variability’) were satisfied by all trials. Items 5 and 6 concerning blinding of subjects and therapists were only satisfied by one trial [21]. All four trials are classed as high quality by achieving greater than five criteria, however, Clarke et al. and Kongsgaard et al. were of particularly high quality, fulfilling 10 and 9 items, respectively.

**Table 4:** Results of PEDro quality assessment Bold and italicised columns indicated for those which influence bias

PEDro Criteria/ Trial	1	2	3	4	5	6	7	8	9	10	11	Total
Van Ark et al. 2018 [11]	✓	✓	X	X	X	X	✓	X	✓	✓	✓	6
Clarke et al. 2011 [21]	✓	✓	✓	X	✓	✓	✓	✓	✓	✓	✓	10
Kaux et al. 2016 [23]	X	✓	X	✓	X	X	X	✓	✓	✓	✓	6
Kongsgaard et al. 2009 [25]	✓	✓	✓	✓	X	X	✓	✓	✓	✓	✓	9

Assessment of the non-randomised observational studies using the Downs and Black scale shown in Table 5, yielded one high quality [20], one moderate quality [24] and three limited quality studies [22,26,27]. The fulfilment of Items 1, 2, 4, and 6 indicates appropriate reporting of the studies, with Item 10’s success indicating adequate reporting of P-values, and Item 18 identifying appropriate statistical analysis. Any items concerning blinding or randomisation of participants or reporters were considered poorly fulfilled, and the frequent occurrence of unable-to-determine (UTD) features did not indicate great study reporting quality.

**Table 5:** Results of Downs and Black quality assessment Bold and italicised columns indicated for those which influence bias

D&B Criterion/ Trial	1	2	3	4	5	6	7	8	9	10	11	12	13	14	15	16	17	18	19	20	21	22	23	24	25	26	Total
Abate et al. 2018 [20]	✓	✓	✓	✓	✓	✓	✓	✓	✓	✓	✓	✓	X	UTD	X	✓	✓	✓	✓	✓	✓	UTD	✓	X	✓	✓	21
Arquer et al. 2014 [27]	✓	✓	✓	✓	X	✓	X	X	X	✓	✓	X	✓	X	X	✓	✓	✓	UTD	X	X	✓	X	X	X	✓	13
Ferrero et al. 2012 [26]	✓	✓	✓	✓	X	✓	✓	✓	X	✓	X	X	X	X	X	✓	✓	✓	✓	X	UTD	UTD	X	X	X	✓	13
Filardo et al. 2013 [22]	✓	✓	X	✓	X	✓	✓	X	✓	✓	X	X	X	X	X	X	X	✓	✓	✓	UTD	UTD	X	X	X	X	10
Kaux et al. 2015 [24]	✓	✓	✓	✓	X	✓	✓	✓	✓	✓	✓	✓	✓	X	X	✓	✓	✓	✓	X	✓	UTD	X	X	X	✓	18

The STROBE statement provided a framework to semi-quantitatively assess the prospective case series (Table 6). The setting of the study along with sources and methods of participant selection were omitted, as well as study size calculations. Also, the number of individuals at each study stage was not elicited, and along with a lack of confounder-adjusted estimates, this impacted the study’s quality. Other than these, the study contained the majority of the relevant features required for it to be determined as moderate quality in the eyes of the assessors.

**Table 6:** Results of STROBE quality assessment

STROBE critera/ trial	1	2	3	4	5	6	7	8	9	10	11	12	13	14	15	16	17	18	19	20	21	22	Total
Gatz et al. 2017 [19]	✓	✓	✓	✓	X	X	X	✓	✓	X	✓	✓	X	✓	✓	X	✓	✓	✓	✓	✓	✓	16

Risk of Bias

The risk of bias was assessed quantitatively in both PEDro and the Downs and Black scale, indicated by the bolded and italicised entries in Tables 3-4, as these are the features which influence bias. Kaux et al. [23] were heavily open to bias due to the lack of blinding of subjects, therapists and assessors without allocation concealment. Clarke et al. [21] showed the strength of their study through blinding adherence, and two studies within the Down’s and Black scale performed particularly poorly [22,26]. Ferrero et al. and Filardo et al. failed to provide details regarding the representative nature of the samples, and no blinding took place in either study. They also failed to carry out randomisation and adjust for confounders within their studies. Criteria 21 to 25 were poorly adhered to upon the Downs and Black scale, exposing the risk of bias within the observational studies it was applied to. In the study by Gatz et al. [19], interventions were not randomised, though assessors were blinded when performing ultra-sonographic tendon assessment. Being a case series it is heavily prone to selection bias with low internal validity due to a lack of control group.

Discussion

Primary Conclusion

This systematic review provides corroborative evidence indicating that changes in ultrasound measures, such as tendon structure and neovascularisation, are not congruent with clinical change in the form of VISA-P. The 10 studies did not exhibit a clear association, indicating that ultrasound tendon change is not responsible for clinical improvement in patients being treated for tendinopathy.

Causes for Limited Association

There are a number of potential reasons behind this lack of association, which may be explained within the wider evidence base surrounding tendinopathy. Firstly, structural changes within a tendon take longer to occur than clinical improvement, therefore follow-up needs to last a significant length of time to map this. Filardo et al. exhibited the longest follow-up at 194 weeks and provided a limited quality association between ultrasound change and VISA-P, however, more evidence may be needed to disprove this. There may be a short-term macromolecular improvement in structure that does not translate to a change in tendon fibrillary alignment and vascularisation [28]. Two high-quality RCTs by Clarke et al. [21] and Kongsgaard et al. [25] are examples of strong evidence indicating a lack of association between VISA-P outcome and ultrasound change. 

Reviewing Trends in Associated Literature

When considering the broader body of evidence associated with other types of tendinopathy, possible reasons become evident. A prospective study of 57 symptomatic Achilles tendons showed that reduced hypo-echo texture did not correlate with clinical outcomes on conventional ultrasonography [29]. Furthermore, the process of neovascularisation has also been indicated to be unrelated to pain [30], therefore when taking in wider tendinopathy evidence the conclusions drawn from this review are not isolated.

Interaction of Structural Characteristics on Selected Outcomes

Another aspect to be considered is that VISA-P is assessing pain and function, and these key domains of tendinopathy may not be influenced by structural characteristics such as thickness and fibrillary alignment, therefore no association would be evident during this study of two unrelated entities.

Limitations

As seen in Table 3, the heterogeneity of scoring for the ultrasound measures leads to inaccuracy in the conclusions that can be drawn from the comparison. Colour Doppler alone is reported in five different ways in the papers assessed, therefore the data produced may not accurately correlate with reality. Additionally, the lack of high-quality studies is a significant limiting factor within this review.

The omission of data in the meta-analysis further limited the conclusions, and for studies such as Abate et al. [20], the semi-quantitative scales they produced had to be altered into means which allowed for further data inaccuracy in reporting in order to formulate them into a format adequate for analysis.

Clinical Implications

From the results of this review, the lack of congruency indicates that ultrasonographic change is not a reliable method of staging a recovery from patellar tendinopathy after intervention. Furthermore, though evidence exists to indicate ultrasound changes predict tendinopathy, ultrasound assessment after clinical diagnosis of patellar tendinopathy is unnecessary as clinical outcomes are the main indicators for return to normal function, independent of structure or vascularization observed on ultrasound. As a result of this, any interventions targeted at changing tendon structure or fibrillar alignment alone rather than clinical outcomes are not effective as treatment goals for patellar tendinopathy.

Further Research Recommendations

There is a clear need for homogeneity of ultrasound measures for tendinopathy, along with universal strategies of reporting these individual measures as the variation between studies allows for significant inaccuracy and reliability of results. More high-quality studies, ideally randomised control trials, are needed to strengthen the limited evidence base available from which the previous conclusions were drawn. Studies which are targeted at evaluating a specific ultrasound outcome measure, in greater volume and consistency, would be the ideal future of research in this area, as well as assessing the variations in ultrasound changes against other PROMs, such as the VAS scale.

## Conclusions

As a highly prevalent condition in young athletes, patellar tendinopathy is a heavily researched area with high clinical relevance but a lack of conclusive evidence to guide practice. Imaging modalities and patient-reported outcome measures such as VISA-P and VAS are extremely valuable in the correct setting and timing when defining patellar tendinopathy, however, this review has concluded that ultrasound measure change is not congruent with a change in VISA-P score after intervention. The lack of congruency may be impacted by low study yield and decreased study quality of those included, therefore increased evidence base assessing more homogenous ultrasound measure reporting will be beneficial for practice. Direct comparison with pain-based measures will be valuable in further research in order to clarify if structural changes, which are slowly occurring, have any impact on short-term symptom alteration. If this is not the case, then the use of ultrasound to specifically assess these domains during follow-up will be inaccurate. 

## Appendices

Appendix 1 (Table 7) covers the search terms used to obtain the data for the study

**Table 7:** Appendix 1 - Search Terms

Search Terms	((Patella* OR Knee*) AND (tendin* OR tendon* OR jumper* OR jumping* OR lander* OR landing*)) AND (Outcome* OR assessment OR instrument* OR scale* OR evaluation* OR questionnaire OR VAS OR visa-p OR blazina scale OR lysholm OR tegner OR SF-36 OR koos OR ostrc OR ikdc OR whoqol-bref OR EQ-5D OR SF-36 OR aqol OR illinois run OR maximum tendon diameter OR tendon proportions OR antero-posterior diameter OR visa-p OR pain numerical rating score OR pain intensity OR cpgs OR knee function OR modified ohberg OR Ultraso* OR torque OR flexibility OR oslo sports trauma OR single-item work ability index OR fabq)
